# Effects of adding unstable-surface balance training to resistance training on balance and agility performance in male college ballet dancers

**DOI:** 10.3389/fphys.2026.1889958

**Published:** 2026-08-24

**Authors:** Lei Yang, Ni Zhen, Ming Li

**Affiliations:** 1 College of Tourism and Wellness, Changzhou Vocational Institute of Industry Technology, Changzhou, Jiangsu, China; 2 Department of dance, Sangmyung University, Seoul, Republic of Korea; 3 Changxin International College of Art, Yunnan University, Kunming, Yunnan, China

**Keywords:** agility performance, ballet dancers, change of direction, resistance training, unstable-surface training

## Abstract

**Background:**

Ballet requires high levels of postural control, coordination, and movement precision. This study examined whether adding unstable-surface balance training to resistance training improves balance and agility performance in college ballet dancers.

**Methods:**

Thirty male college ballet dancers were randomly assigned to an unstable-surface combined training group (CT, n = 15) or a stable-surface comparison group (ST, n = 15). Both groups completed a 12-week training program, with three sessions per week. Each session comprised 40 min of resistance training and 20 min of balance training. Static balance, dynamic balance, sensory organization, Y-Balance Test performance, and change-of-direction outcomes were assessed before and after the intervention.

**Results:**

Significant time effects and group × time interactions were observed for all static-balance variables under the eyes-open and eyes-closed conditions (all p < 0.05), with broader improvements in the CT group than in the ST group. Significant time effects and group × time interactions were also observed for VIS, VEST, toes-up and toes-down adaptation, YBT-N, YBT-D, and closed-eye stepping performance (all p < 0.05). No significant time effects or group × time interactions were found for the SOT composite score, SOM, or PREF. All three change-of-direction outcomes improved over time, but none showed a significant group × time interaction.

**Conclusions:**

Unstable-surface balance training combined with resistance training produced broader improvements in static and dynamic balance than stable-surface balance training. Evidence for superior agility improvement, however, remained limited.

## Introduction

Ballet is a physically demanding activity that requires high levels of postural control, coordination, and movement precision, with balance serving as a fundamental component of successful performance (Hrysomallis, 2011). Ballet dancers must frequently maintain equilibrium during single-leg support, asymmetrical postures, rapid transitions, and rotational movements while preserving technical quality and aesthetic form (Hrysomallis, 2007). These demands require effective integration of proprioceptive, neuromuscular, visual, vestibular, and somatosensory inputs for movement execution and injury prevention. Indeed, this load is reflected in a high injury burden: musculoskeletal injury prevalence in ballet dancers exceeds 80%, lower-extremity injuries account for approximately 66–91% of all injuries, and the foot and ankle are the most commonly affected region (14–57% of injuries; the ankle alone 16–33%), with reported injury incidence rates of 0.97–1.24 per 1000 dance-hours (Critchley et al., 2022; Smith et al., 2015). Impaired postural control and ankle instability are recognized contributors to this elevated risk (Hong et al., 2025). Accordingly, training strategies that further enhance balance-related performance are of considerable interest in ballet education and conditioning practice.

Although regular ballet practice can improve balance and coordination, supplementary conditioning may provide benefits beyond ballet-specific training alone. Resistance training (RT) has been shown to enhance muscular strength, power, and movement capacity, which may contribute to improved dynamic postural control and more effective responses to perturbations (Giagazoglou et al., 2013; Sekulic et al., 2013). In parallel, unstable-surface training (UST), performed on devices such as BOSU balls, Swiss balls, and balance pads, has been widely used to increase postural demands and challenge neuromuscular regulation (Cao et al., 2025; Silva et al., 2024). By requiring continuous adjustments to maintain equilibrium, UST may promote adaptations that are particularly relevant for ballet dancers, whose movements often involve narrow bases of support, single-leg stances, and dynamic turns (Giagazoglou et al., 2013). In dancers specifically, randomized and controlled neuromuscular and proprioceptive training interventions have improved postural control and dynamic balance and have been associated with reduced lower-limb injury risk (Mancini et al., 2025; M. Zhang et al., 2021), indicating that supplementary balance-oriented conditioning can yield measurable benefits even in trained dance populations.

From an applied perspective, combining RT with balance training may be particularly beneficial for ballet dancers. RT may provide the force-generating capacity required for movement execution, whereas balance training may improve the control and coordination needed to use that force effectively during complex motor tasks (Granacher and Behm, 2023). Previous studies have examined combined training approaches in athletic populations; however, evidence remains limited on whether adding unstable-surface balance training to RT provides greater benefits than performing the same balance exercises on stable surfaces in college ballet dancers. This distinction is important because ballet dancers typically have relatively high baseline balance ability, and it remains unclear whether a more challenging postural environment can elicit additional functional gains in this population. To date, most combined- and unstable-surface training studies have been conducted in general athletes, recreationally active adults, or older populations rather than dancers, and the few dancer-based studies have not held the resistance-training stimulus and balance exercises constant across surface conditions; the independent contribution of surface instability therefore remains unclear. By keeping the resistance-training stimulus and the balance exercises identical across groups, the present study isolates the effect of surface instability itself and provides practically meaningful guidance on whether the added complexity and potential risk of unstable-surface training are justified in this high-baseline-balance population.

Change-of-direction performance is also relevant in ballet, particularly during sequences that require rapid transitions between rotational and lateral movements. These tasks depend on the integration of strength, coordination, and dynamic postural control (Sekulic et al., 2013). Therefore, examining both balance and agility-related outcomes may provide a more comprehensive understanding of the functional effects of supplemental conditioning in ballet dancers.

Accordingly, the object of this study was to compare the effects of 12 weeks of RT combined with either unstable-surface or stable-surface balance training on static balance, dynamic balance, and agility performance in college ballet dancers. We hypothesized that, relative to the stable-surface comparison group, the unstable-surface training group would achieve significantly greater improvements in balance performance and would show greater gains in selected dance-relevant agility tasks.

## Materials and methods

This study employed a randomized controlled, parallel-group design. The target population consisted of college ballet dancers enrolled in undergraduate dance programs. Participants were recruited using convenience sampling based on their availability and willingness to participate. An *a priori* power analysis was conducted using G*Power software (version 3.1.9.7) to determine the required sample size. Based on previous research examining the effects of unstable-surface training on balance, an effect size (Cohen’s f) of 0.25 was anticipated. With an alpha level of 0.05 and statistical power of 0.80, the analysis indicated that at least 24 participants, with 12 participants per group, were required. Considering potential attrition, the recruitment target was increased to 30 participants. Thirty eligible college ballet dancers were enrolled, all of whom were male.

The inclusion criteria were as follows: (1) aged 18–23 years; (2) currently enrolled as a college ballet dancer and participating in regular ballet training; (3) able to complete all testing and training sessions throughout the intervention period; and (4) willing to provide written informed consent before participation. The exclusion criteria were as follows: (1) a history of severe lower-extremity injury within the previous three years, including anterior cruciate ligament, hamstring, meniscus, or ankle injury; (2) any medical, neurological, or orthopedic condition that could interfere with balance performance, change-of-direction assessment, or participation in the training program; and (3) inability to complete the prescribed intervention or testing procedures.

The study was approved by the Shandong Normal University Ethics Committee (Approval number: SDNUTYDW2025050) and adhered to the latest revision of the Declaration of Helsinki. Before enrollment, all participants were fully informed about the study procedures, potential risks, and benefits, and written informed consent was obtained. This study was not registered in a publicly accessible trial registry before or after participant recruitment. The absence of trial registration is acknowledged as a methodological limitation.

### Procedures

**Figure 1:**
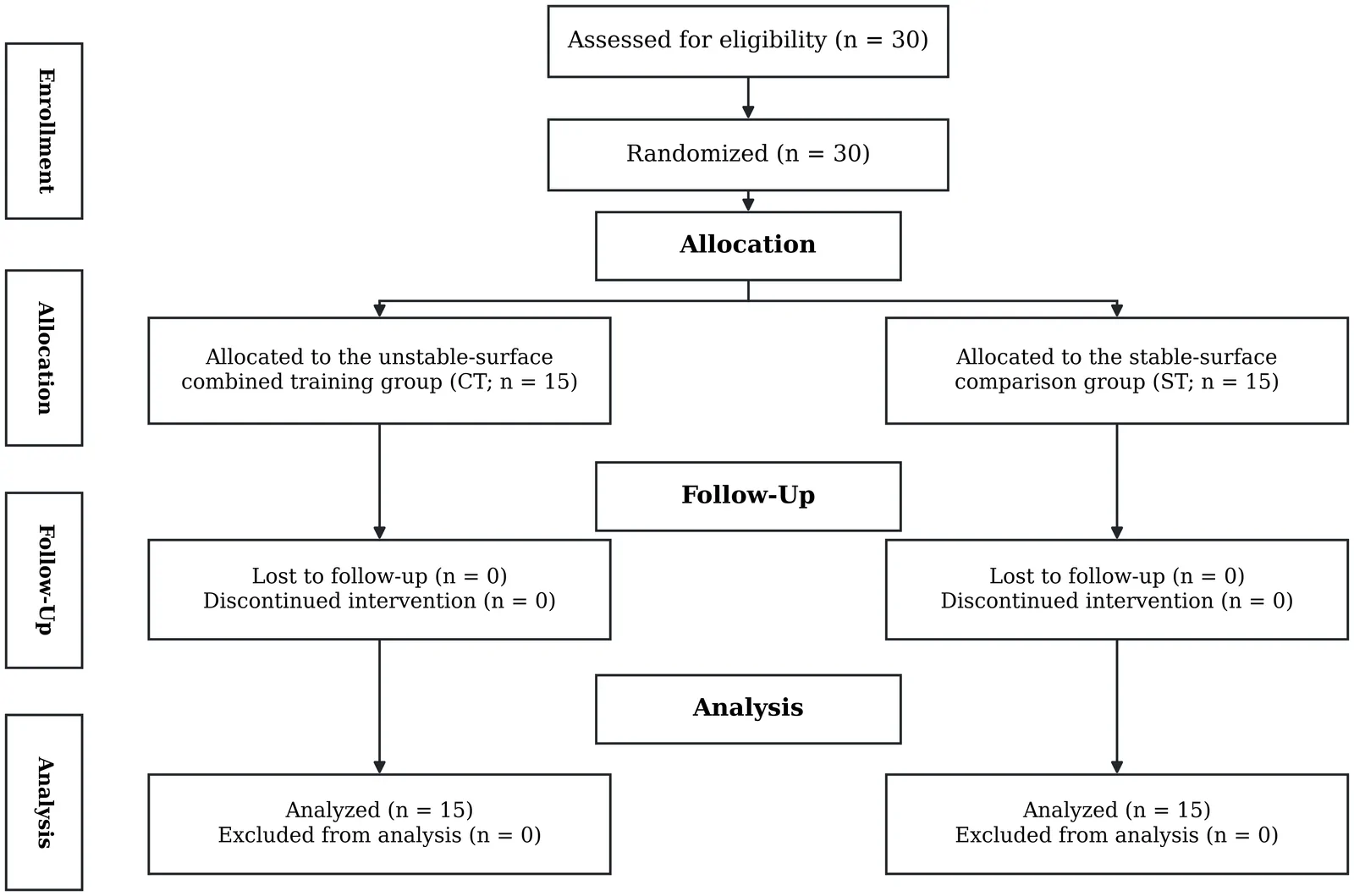
Flow chart of participant progress through the study phases according to the CONSORT statement.

The 30 male participants were randomly allocated to either the unstable-surface combined training group (CT, n = 15) or the stable-surface comparison group (ST, n = 15) using a computer-generated randomization list (**Table 1**). All outcome assessments were performed by independent assessors who were blinded to group allocation (**Figure 1**). Standardized verbal encouragement was provided during testing to support consistent effort across participants. Before the intervention, all participants completed a 2-week familiarization period (three sessions per week) to become accustomed to the testing procedures and training exercises. During the 12-week intervention, both groups trained three times per week, with 48 h of recovery between sessions. The CT group performed the balance training program on unstable surfaces (**Table 2**), whereas the ST group performed the same balance exercises on stable surfaces. Both groups completed the same resistance training program (**Table 3**).

**Table 1:** Physical characteristics of participants in the CT and ST groups.

Group	Age(y)	Height(cm)	Mass(kg)
CT(n=15)	21.65 ± 2.68	178.33 ± 3.52	67.80 ± 4.82
ST(n=15)	21.40 ± 2.56	178.56 ± 5.73	72.02 ± 5.99

**Table 2:** The main part of the balance training program.

Exercise	Block 1(week1-4)	Block 2(week5-8)	Block 3(week9-12)
Balance Board Exercise	Static standing on the board with two legs (30 s/set*3set)	Static standing on the board with two legs and eyes closed (30 s/set*3set)	Squat on the plate with eyes closed (10 reps/set*3set)
Swiss Ball Exercise	Isometric supine straight leg bridge on Swiss Ball (30 s/set*3set)	Isometric supine single-leg bending bridge on Swiss Ball (30 s/set*3set)	Dynamic supine single-leg bending bridge on Swiss (10 reps/set*3set)
Side-plank with Inflated Balance disc Exercise	Side-plank with inflated balance disc with elbow (30 s/set*3set)	Side-plank with inflated balance disc and the non-supporting leg stretches backward (30 s/set*3set)	Side-plank with inflated balance disc and the non-supporting leg stretches backward with elastic band (10 reps/set*3set)
Bosu Ball Exercise	Lunge squat on BOSU ball (10reps/leg/set*3set)	Lunge squat on BOSU ball and inflated balance disc (10reps/leg/set*3set)	Lunge squat on BOSU ball and inflated balance disc with 5 kg dumbbells (10reps/leg/set*3set)
Airex® Balance-pad Elite Exercise	Single-leg squat with balance-pad (10reps/leg/set*3set)	Single-leg standing with balance-pad and the non-supporting leg stretches backward (12 reps/leg/sets*3set)	Single-leg support with balance-pad elite and the non-supporting leg stretches backward with elastic band (12 reps/leg/sets*3set)
Rest	Between exercise: 60 s Between sets: 3 min		

CT group conducted training program on unstable support (e.g., BOSU ball, Swiss ball, and Balance pad); and ST group conducted training program on stable support (i.e., solid floor).

**Table 3:** The main part of the resistance training program.

Exercise	Block 1(week1-4)	Block 2(week5-8)	Block 3(week9-12)
Single Leg	Single Leg Squat (10reps/leg/set*3set)	Single Leg Squat (12reps/leg/set*3set)	Single Leg Squat (12reps/leg/set*4set)
Upper Body	Push Ups (20reps/set*3set)	chin-up (20reps/set*3set)	chin-up (20reps/set*4set)
Lower Body	Barbell Deadlift (10reps/set*3set) Back Squat (10reps/set*3set)	Barbell Deadlift (4reps/set*5set) Back Squat (4reps/set*5set)	Barbell Deadlift (3reps/set*3set) Back Squat (3reps/set*3set)
Pull	Single Arm Row (10reps/side/set*3set)	Single Arm Row (12reps/side/set*3set)	Single Arm Row (12reps/side/set*4set)
Posterior Chain	Lateral Split Squat (10reps/leg/set*3set)	Single Leg Romanian Deadlift (10reps/leg/set*3set)	Single Leg Romanian Deadlift (12reps/leg/set*4set)
Power		Hang Clean (3reps/set*4set)	Hang Clean (3reps/set*6set)
Intensity	65% 1RM	75% 1RM	85% 1RM
Rest	Between sets: 3 min		
Dynamic movements in a yoga-flow pattern were performed prior to each training session. Squatting, lunging, quadruped, shoulder mobility, foam rolling, resistance band terminal knee extension, hip external rotator and abductor muscle activation exercises against gravity Duration: 5 min			

Training loads for the primary externally loaded resistance exercises were individualized according to the 1RM values assessed at the beginning of each four-week block. Target intensity increased from 65% 1RM in Block 1 to 75% 1RM in Block 2 and 85% 1RM in Block 3. The number of repetitions was progressively reduced as training intensity increased. The prescribed intensity did not exceed 85% 1RM. Bodyweight and accessory exercises were prescribed according to the sets and repetitions shown in the table rather than as a percentage of 1RM.

### Training program

The intervention protocol was developed specifically for the present study and tailored to the physical demands of male collegiate ballet dancers rather than being reproduced verbatim from a single previously published program. Its design was informed by established resistance-training principles and previous resistance and neuromuscular training interventions conducted in dancers and physically active populations. Both groups completed 36 supervised training sessions over 12 weeks, with three sessions performed each week and at least 48 h of recovery between consecutive sessions. Each session consisted of approximately 40 min of resistance training followed by 20 min of balance training.

The resistance-training component included bilateral and unilateral lower-extremity exercises, upper-body pushing and pulling exercises, posterior-chain exercises, and a power-oriented exercise. Back squats and barbell deadlifts were selected to develop general hip- and knee-extensor strength and posterior-chain force capacity. Single-leg squats, lateral split squats, and single-leg Romanian deadlifts were included to develop unilateral force control, pelvic stability, frontal-plane control, and lower-extremity alignment, which are relevant to single-leg support, turnout positions, landings, and directional transitions in ballet. Push-ups, chin-ups, and single-arm rows were included to support upper-body strength and whole-body postural control, whereas the hang clean was incorporated to emphasize rapid force production and coordinated whole-body extension. The relevance of the single-leg squat to ballet is supported by evidence that single-leg squat performance is associated with lower-extremity alignment during ballet movements performed in turnout (Hopper et al., 2016).

The resistance-training program followed a linear progression model consisting of three consecutive four-week blocks. Loads for the primary externally loaded exercises were individualized according to 1RM values assessed at the beginning of each block. The target intensity progressed from 65% 1RM in Block 1 to 75% 1RM in Block 2 and 85% 1RM in Block 3, while the prescribed repetitions for the primary strength exercises decreased from 10 to 4 and then to 3 repetitions per set. The first block emphasized movement competency and tolerance to training volume, the second block emphasized strength development, and the final block emphasized higher-force strength performance. Training intensity did not exceed 85% 1RM. This progression was consistent with established resistance-training principles recommending periodized exposure to moderate-to-heavy loads, the inclusion of bilateral and unilateral multi-joint exercises, and progressive load adjustment according to the participant’s ability to complete the prescribed repetitions with correct technique (NA, 2009).

The 12-week duration and three-session-per-week frequency were selected to provide a sufficient training stimulus while allowing recovery and minimizing interference with regular ballet training. Previous dancer-specific interventions have shown that supplementary resistance training conducted for approximately 9–12 weeks can improve lower-extremity strength, power, dynamic balance, and dance-related performance (Dowse et al., 2017). Similarly, a 10-week neuromuscular training intervention performed three times per week improved postural-control outcomes in competitive dancers (Meiqi Zhang et al., 2021).

The balance-training program was designed to progressively increase postural, neuromuscular, and sensory demands across the three four-week blocks. Balance boards, Swiss balls, BOSU balls, inflated balance discs, and Airex balance pads were selected because they allowed systematic manipulation of support-surface stability, base of support, visual input, unilateral loading, movement complexity, and external resistance. Progression was achieved by moving from bilateral to unilateral support, from eyes-open to eyes-closed conditions, from static to dynamic exercises, and from bodyweight tasks to exercises incorporating dumbbells or elastic resistance. These progressions were considered relevant to ballet because dancers frequently perform under conditions involving single-leg support, narrow bases of support, rotational movement, and continuous postural adjustment. Previous interventions using foam pads, BOSU balls, balance discs, single-leg tasks, squats, and lunges have demonstrated improvements in postural control and dynamic balance in dancers and physically active young adults (Meiqi Zhang et al., 2021).

The CT and ST groups completed identical resistance exercises and identical balance exercises, with the same exercise order, sets, repetitions, external loads, training duration, and recovery intervals. The only systematic difference between groups was the support-surface condition used during balance training: the CT group performed the exercises on unstable surfaces, whereas the ST group performed the same exercises on stable surfaces. This matched active-comparison design was used to isolate the specific contribution of surface instability. During the intervention period, all participants continued the same institutionally scheduled ballet curriculum, comprising approximately 12 h of ballet training per week. Because participants were recruited from the same academic program, scheduled ballet-training exposure was equivalent between groups. Participants were instructed not to undertake additional resistance, balance, or structured neuromuscular training outside the study intervention. Compliance with this instruction was assessed through weekly verbal confirmation.

### Assessment protocols for static and dynamic balance

#### Assessment procedures overview

All outcome assessments were conducted before and after the 12-week intervention under standardized laboratory conditions. Participants were instructed to avoid strenuous exercise for 24 h before testing and maintain their habitual dietary and hydration habits. All post-intervention assessments were performed at the same time of day as the baseline assessment whenever possible to minimize the influence of circadian variation. Before each assessment session, participants completed a standardized warm-up consisting of 15 min of low-intensity activity and dynamic movements. The order of assessments was identical at baseline and post-intervention testing. A trained assessor provided standardized instructions before each test, and the same assessor recorded all performance outcomes. For tests requiring repeated trials, participants completed three valid attempts, and the best performance or mean value was retained according to the predefined scoring criteria.

#### Static balance assessment

Static balance was assessed using a force-plate system (Good Balance Static Balance Tester, Metitur, Finland) during a 30-s single-leg stance on the dominant leg, defined as the participant’s self-reported preferred kicking leg. Testing was performed under eyes-open conditions, with a fixed forward gaze and the contralateral limb held 20–30 cm anterior to the platform, and under eyes-closed conditions. Center-of-pressure (COP) sway was quantified separately in the mediolateral (X) and anteroposterior (Y) directions using sway displacement and mean COP velocity. Force-platform-derived center-of-pressure displacement and mean velocity were selected as objective indicators of postural steadiness. The construct validity of center-of-pressure-based measures is supported by their sensitivity to established differences associated with age and visual availability during quiet standing (Prieto et al., 1996). In single-leg stance testing, center-of-pressure measures have demonstrated moderate-to-excellent test–retest reliability, with velocity-based variables generally showing greater consistency than displacement-based variables (Kozinc et al., 2025).

### Dynamic balance assessment

#### Y-balance test (FMS Kit, Move2Perform, USA)

Dynamic balance was evaluated using the Y-Balance Test. Participants stood on one limb while reaching maximally in three directions: anterior, posteromedial, and posterolateral. Three trials were completed in each direction, and reach distance was recorded to the nearest 0.5 cm. A normalized composite score (%) was calculated as follows:


Composite score(%)=[(anterior+posteromedial+posterolateral reach distances)/(3×limb length)]×100

Limb length was measured using a standardized procedure. For clarity, YBT-N denotes the normalized composite score, whereas YBT-D denotes the dominant-limb score reported in the results tables. The Y-Balance Test was selected as a standardized measure of dynamic postural control. The instrumented Y-Balance device has demonstrated good intrarater reliability (ICC = 0.85–0.91) and excellent inter-rater reliability (ICC = 0.99–1.00) for the three reach directions (Plisky et al., 2009). Comparative testing against the Star Excursion Balance Test has provided partial convergent evidence for the posteromedial and posterolateral reach directions, although anterior-reach scores differ between the two procedures and should not be considered interchangeable (Coughlan et al., 2012). More recent evidence has also demonstrated good inter-rater and test–retest reliability for the reach-direction and composite scores (ICC ≈ 0.79–0.86) (Foldager et al., 2023).

#### Closed-eye in-place stepping test

This protocol was adapted from the Fukuda/Unterberger stepping paradigm; however, the present outcome—time to first boundary exit from a 40-cm-diameter circle—differs from the conventional angular-rotation and linear-displacement outcomes. Previous studies of the conventional Fukuda Stepping Test have reported variable test–retest reproducibility and limited sensitivity and specificity for identifying unilateral vestibular dysfunction (Zhang and Wang, 2011). Because these measurement properties cannot be directly generalized to the present time-to-boundary-exit outcome, this variable was treated as an exploratory indicator of closed-eye stepping stability. The mean of three standardized trials was used to reduce random measurement error.

### Computerized dynamic posturography

CDP was conducted on the Bertec Balance Advantage system, with participants barefoot and secured in a safety harness. The Sensory Organization Test (SOT) comprised six sensory conditions that combined fixed versus sway-referenced visual-surround and support-surface inputs; three 20-s trials were completed for each condition. Outcomes included the composite score and sensory ratios (SOM, VIS, VEST, PREF). The Sensory Organization Test was selected because it systematically manipulates the availability and accuracy of visual and support-surface information, thereby assessing the relative use of somatosensory, visual, and vestibular inputs for postural control. Evidence of known-groups and construct validity is provided by the differentiated postural responses observed in individuals with vestibular deficits under altered support and visual conditions (Nashner et al., 1982). Test–retest reliability has been demonstrated for Sensory Organization Test outcomes (Ford-Smith et al., 1995), although repeated administration may produce learning effects in healthy young adults. On the Bertec platform, acceptable-to-good reliability has been reported for Sensory Organization Test outcomes (ICC ≈ 0.73–0.84) (Lu et al., 2025); therefore, familiarization and standardized testing procedures were used in the present study.

### Adaptation test

The Adaptation Test was selected to quantify adaptive postural responses to repeated support-surface perturbations. Its construct rationale is supported by experimental evidence showing that repeated platform rotations elicit progressive adaptation of automatic postural responses (Nashner et al., 1982). This response pattern supports the construct validity of using repeated toes-up and toes-down rotations to assess adaptive postural control. The sway-energy score reflects the magnitude of the corrective postural response required following each perturbation, with lower values indicating more efficient adaptation. Moderate test–retest reliability has been reported for Adaptation Test sway-energy outcomes on the Bertec platform (ICC ≈ 0.77) (Lu et al., 2025).

### Change of direction test

#### Post-rotation lateral change-of-direction agility test

A ballet-specific post-rotation lateral COD test was used to quantify coordination, direction-switch efficiency, and rotational stability during a rapid lateral change of direction immediately after turning. Cones or barrels were arranged in a T-configuration, with 1 m from the start point to the front, right, left, and back markers. From the start, each dancer performed two consecutive flat turns (pirouette en dedans), executed a right lateral pas de bourrée slide to touch the right marker, returned to the start, performed a left slide to touch the left marker, and returned to the start. Total time was recorded from turn initiation to completion of the left-marker touch. This test was developed for the present study to represent preplanned lateral change-of-direction performance immediately following ballet-specific rotational movement. Its content relevance was based on the inclusion of pirouette en dedans, pas de bourrée, turnout, relevé, head-control, and knee-extension requirements that are characteristic of ballet movement. Pilot testing demonstrated good test–retest reliability (ICC = 0.88). However, because formal construct and criterion validity have not yet been established, the outcome was interpreted as an exploratory indicator of ballet-related post-rotation change-of-direction performance rather than a validated measure of overall ballet performance or reactive agility.

#### Ballet multi-directional change-of-direction agility test

The BMDAT quantified multidirectional COD speed under ballet-specific aesthetic constraints. Markers were arranged in a T-configuration with 1.5 m spacing from the start to the front, right, left, and back markers, and dancers wore standardized pointe shoes. From the start, participants (i) touched the front marker via an arabesque glide, (ii) moved laterally to the right to touch the right marker via pas de bourrée, (iii) returned and moved laterally to the left to touch the left marker, and (iv) returned to touch the rear marker, while maintaining relevé and prescribed artistic form, including turnout, extension, épaulement control, and single-leg support in arabesque stance at each touch. The Ballet Multi-Directional Change-of-Direction Test was developed for the present study to assess preplanned multidirectional movement performance under ballet-specific technical and aesthetic constraints. Its content relevance was based on the inclusion of arabesque, pas de bourrée, relevé, turnout, extension, épaulement control, single-leg support, and pointe-shoe conditions. Pilot testing demonstrated excellent test–retest reliability (ICC = 0.91), with a coefficient of variation below 5%. However, formal construct and criterion validity have not yet been established. Therefore, this outcome was interpreted as an exploratory indicator of ballet-related multidirectional change-of-direction performance rather than a validated measure of overall ballet performance or reactive agility.

#### Hexagon agility test

**Figure 2:**
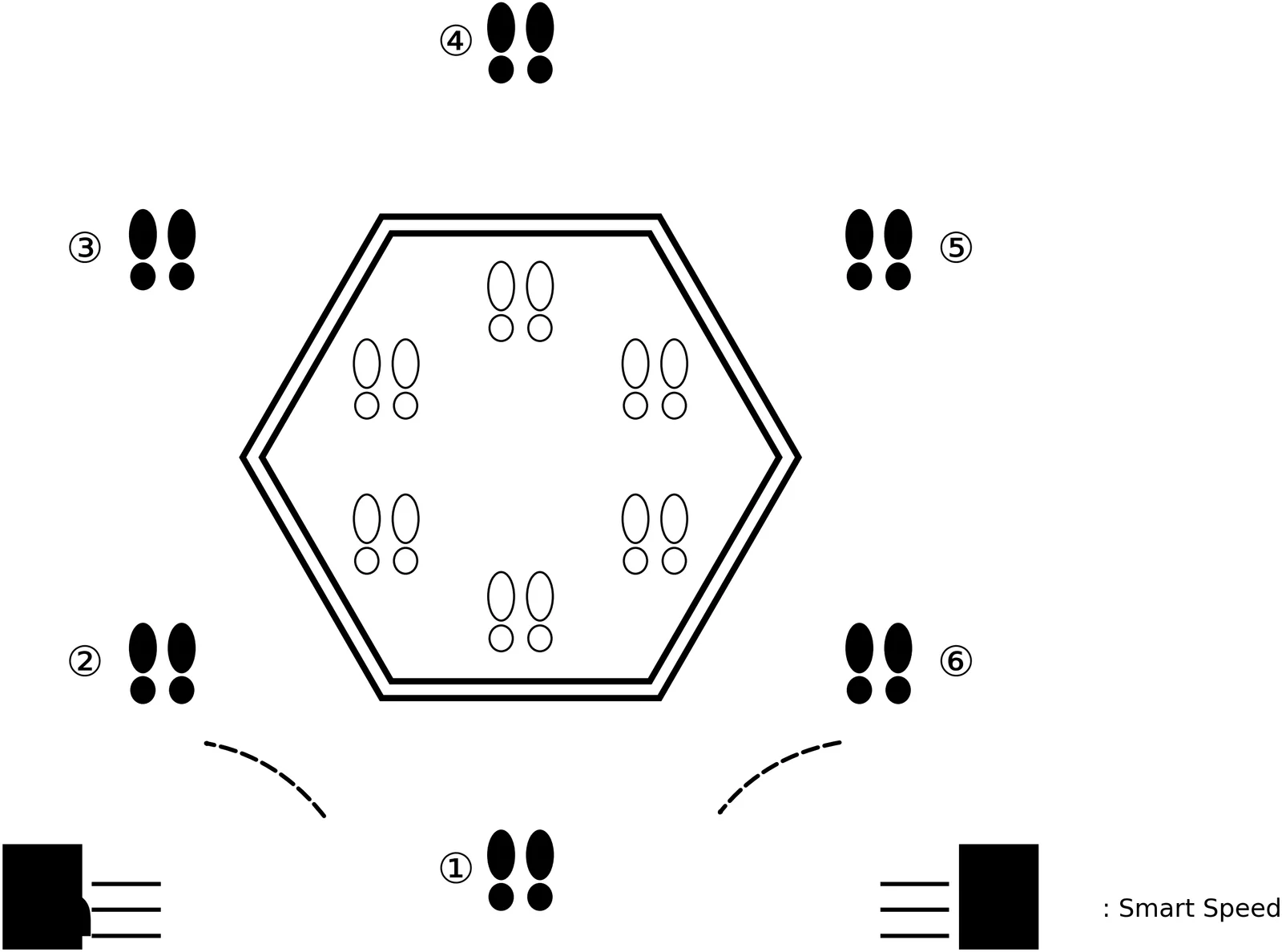
Hexagon agility test design.

General COD ability was assessed using the hexagon agility test (Beekhuizen et al., 2009). Participants started 50 cm behind side 1 of the hexagon and, on the “go” signal, completed rapid jumps into and out of the hexagon in a clockwise sequence (1→6) (**Figure 2**). Three trials were performed with 2 min of passive recovery, and the fastest time was retained. General multidirectional hopping performance was assessed using the Hexagon Test. Beekhuizen et al. (2009) reported excellent same-day and between-day test–retest reliability, with ICC(3,1) values of 0.938 and 0.924, respectively, supporting the reproducibility of the test. However, because the test follows a predetermined movement sequence and does not include a stimulus–response or decision-making component, it was interpreted as a measure of preplanned multidirectional hopping and change-of-direction performance rather than reactive agility. Evidence regarding its criterion validity as a measure of overall agility remains limited.

For tests involving repeated attempts, outcomes were calculated as the mean of three valid trials unless otherwise specified. Trials were repeated and labeled invalid when predefined execution criteria were violated, including loss of balance, additional limb support, an incorrect pathway or cadence, or failure to contact the required markers.

### Statistical analysis

All statistical analyses were performed using IBM SPSS Statistics (version 25.0; IBM Corp., Chicago, IL, USA). Continuous variables are presented as mean ± standard deviation (SD). For each outcome, a two-way mixed repeated-measures ANOVA was conducted, with group (CT vs. ST) as the between-subject factor and time (pre vs. post) as the within-subject factor. Main effects of group and time and the group × time interaction were examined. Normality of residuals was assessed using the Shapiro-Wilk test and visual inspection of Q-Q plots, and homogeneity of variance was examined using Levene’s test. Because the within-subject factor included only two time points, the sphericity assumption was not applicable.

When a significant group × time interaction was identified, Bonferroni-adjusted *post hoc* comparisons were performed to examine pre-post changes within each group and between-group differences at each time point. To reduce the risk of type I error from multiple related outcomes, the false discovery rate (FDR; Benjamini-Hochberg procedure) was applied within each outcome family: static balance, dynamic balance/CDP, and agility/COD outcomes. Effect sizes for ANOVA terms are reported as partial eta-squared (ηp²), and standardized mean differences (Cohen’s d) are reported for pre-post changes. Cohen’s d was calculated as the standardized pre–post difference. Positive values indicate a reduction from pre- to post-intervention, whereas negative values indicate an increase. Consequently, the interpretation of the sign depended on whether higher or lower values represented better performance for the specific outcome. Cohen’s d was interpreted as trivial (<0.20), small (0.20–0.49), moderate (0.50–0.79), and large (≥0.80). Statistical significance was set at p < 0.05.

## Results

All 30 male participants completed the intervention and were included in the final analysis, with 15 participants in each group. No participant withdrew from the study. The principal finding was that CT produced broader improvements than ST in static balance and dynamic balance outcomes, particularly in mediolateral sway control, sensory organization, Y-Balance performance, and closed-eye stepping performance. In contrast, agility-related outcomes improved mainly as a function of time, and between-group superiority of CT over ST was not consistently supported. The CT and ST groups each consisted of 15 male dancers. Because all participants were male, no sex-stratified or between-group sex comparison was applicable. All variables were normally distributed (p > 0.533). No significant between-group differences were observed at baseline for demographic characteristics (age, body mass, and height) or any outcome variable (p > 0.758), indicating that the two groups were comparable before the intervention.

### Static balance

**Figure 3:**
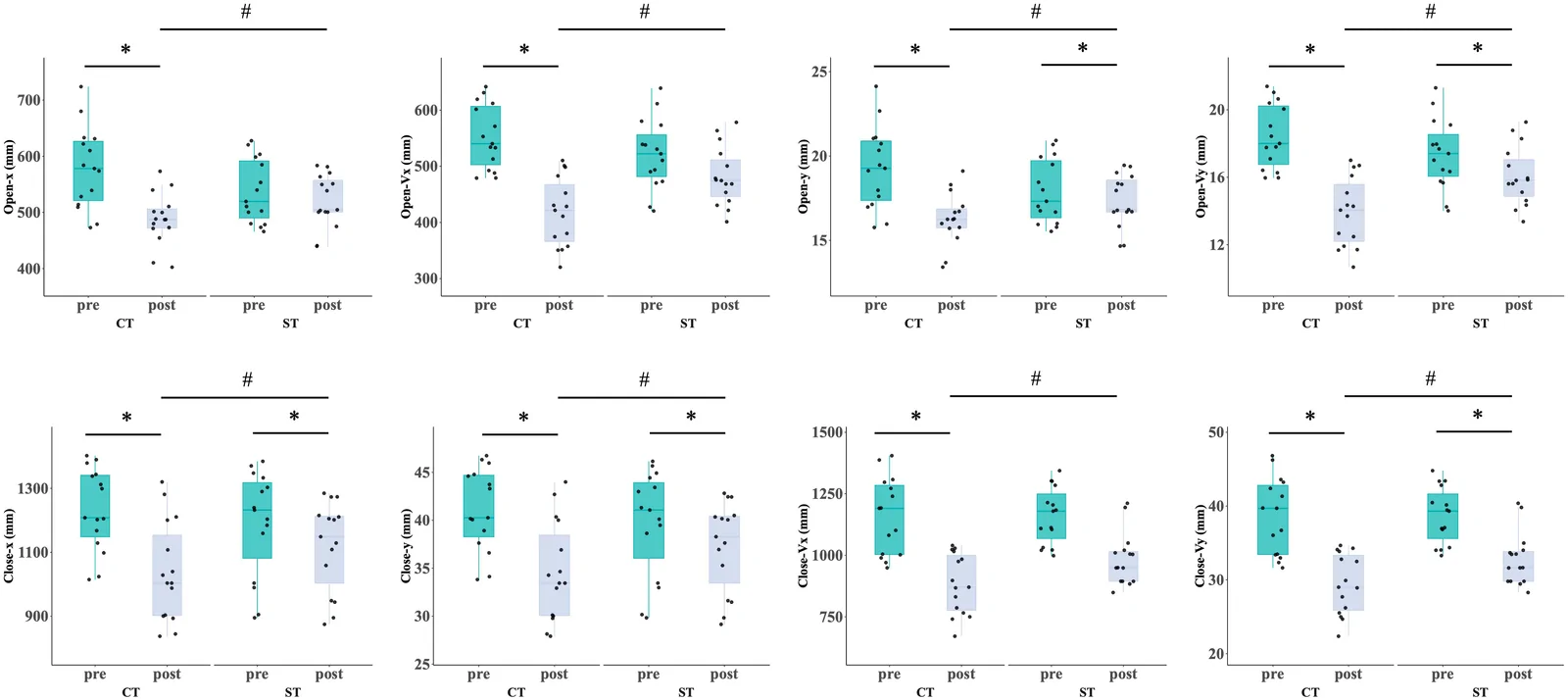
Changes in static balance performance before and after 12 weeks of training in the CT and ST groups. Open-x and Close-x represent mediolateral center-of-pressure (COP) displacement under eyes-open and eyes-closed conditions, respectively. Open-y and Close-y represent anteroposterior COP displacement. Open-Vx and Close-Vx represent mediolateral COP velocity, whereas Open-Vy and Close-Vy represent anteroposterior COP velocity. Values are expressed as mean ± standard deviation. Boxes represent the interquartile range, horizontal lines indicate medians, whiskers represent data distribution, and black dots indicate individual participant values. *Indicates a significant within-group pre–post difference (p < 0.05). ^#^Indicates a significant between-group difference at post-intervention (p < 0.05). CT, unstable-surface combined training group; ST, stable-surface comparison group; COP, center of pressure.

For static balance outcomes, significant main effects of time and significant group × time interactions were observed for Open-x, Open-Vx, Open-y, Open-Vy, Close-x, Close-Vx, Close-y, and Close-Vy (all p < 0.05; **Table 4**; **Figure 3**). *Post hoc* analyses showed that the CT group had significant pre-post reductions in all static balance variables (all adjusted p < 0.001), indicating improved postural stability under both eyes-open and eyes-closed conditions. In contrast, the ST group showed significant improvements only in Open-y (p < 0.001), Open-Vy (p < 0.001), Close-x (p = 0.021), Close-Vx (p = 0.021), and Close-Vy (p < 0.001). Overall, these findings indicate that adding unstable-surface balance training was associated with broader improvements in static postural control than performing the same balance exercises on stable surfaces.

**Table 4:** The static balance assessment results for CT group and ST group before and after 12-week training.

Outcome	CT group			ST group			Time effect	Time × group interaction effect
	Pre	Post	Cohen’s d (95%CI)	Pre	Post	Cohen’s d (95%CI)		
Open-x (mm)	578.55 ± 72.82	488.55 ± 45.93^*#^	1.588 (0.749, 2.427)	537.25 ± 56.87	520.24 ± 46.90	0.300 (-0.307, 0.907)	<0.001	<0.001
Open-Vx (mm/s)	19.29 ± 2.43	16.29 ± 1.53^*#^	1.587 (0.748, 2.425)	17.91 ± 1.90	17.34 ± 1.56	0.301 (-0.306, 0.909)	<0.001	<0.001
Open-y (mm)	552.53 ± 57.25	418.03 ± 61.59^*#^	2.297 (1.377, 3.218)	521.24 ± 62.45	481.59 ± 52.32^*^	0.677 (0.275, 1.080)	<0.001	<0.001
Open-Vy (mm/s)	18.42 ± 1.91	13.93 ± 2.01^*#^	2.297 (1.377, 3.217)	17.37 ± 2.08	16.05 ± 1.74^*^	0.677 (0.275, 1.080)	<0.001	<0.001
Close-x (mm)	1234.05 ± 129.70	1031.37 ± 155.91^*#^	1.320 (0.670, 1.969)	1189.27 ± 266.26	1117.98 ± 141.71^*^	0.478 (0.021, 0.936)	<0.001	<0.001
Close -Vx (mm/s)	41.13 ± 4.33	34.58 ± 5.20^*#^	1.319 (0.670, 1.968)	39.64 ± 5.54	37.27 ± 4.73^*^	0.478 (0.021, 0.935)	<0.001	<0.001
Close -y (mm)	1159.23 ± 156.19	882.95 ± 122.80^*#^	2.183 (1.234, 3.133)	1160.70 ± 114.84	983.72 ± 106.72	1.399 (0.685, 2.112)	<0.001	0.003
Close-Vy (mm/s)	38.64 ± 5.21	29.43 ± 4.09^*#^	2.184 (1.231, 3.134)	38.69 ± 3.83	32.79 ± 3.56^*^	1.399 (0.685, 2.113)	<0.001	0.003

^*^Statistically significant difference between pre- and post-test, P < 0.05.

^#^Statistically significant difference between CT group and ST group, P < 0.05.

### Dynamic balance

**Figure 4:**
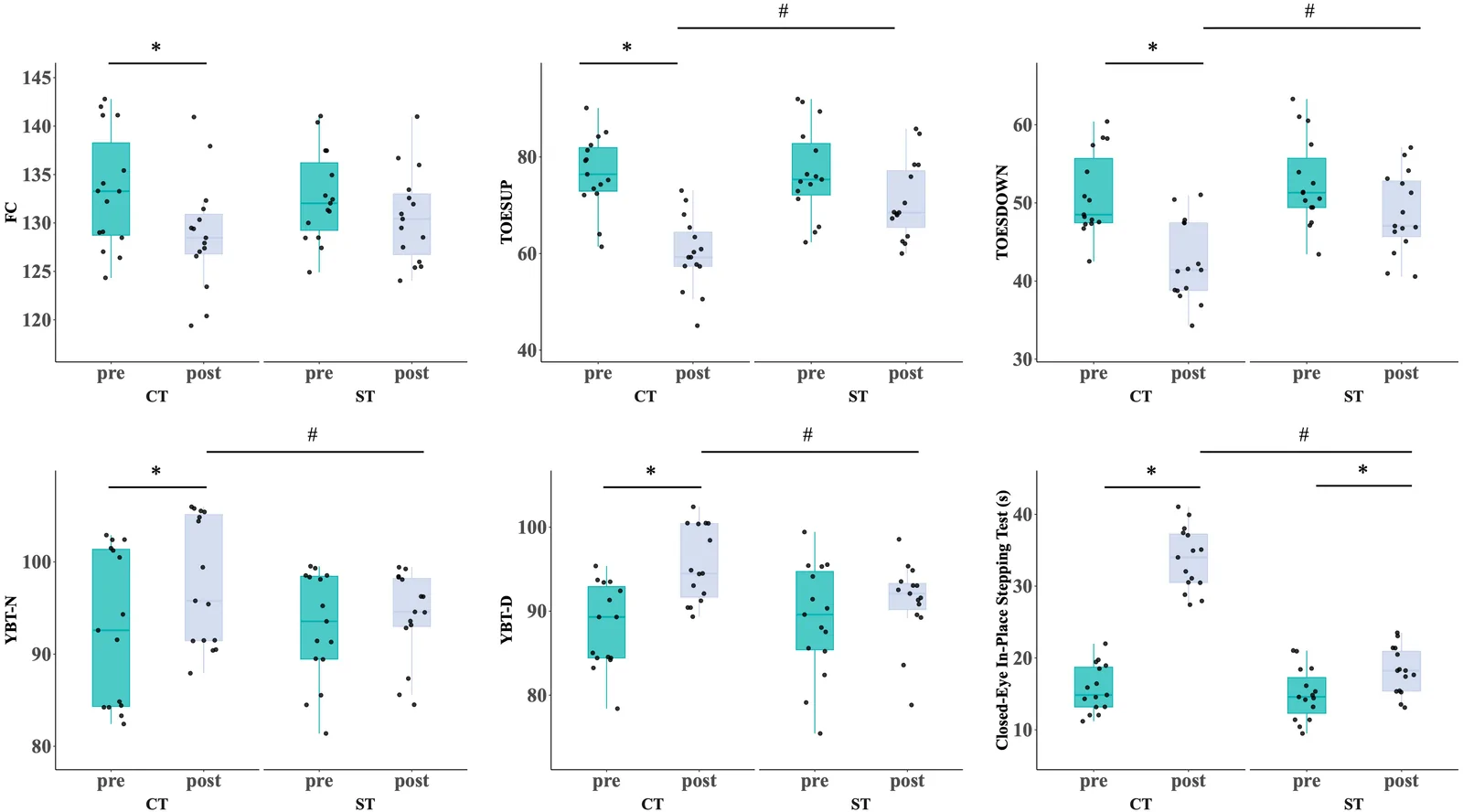
Effects of training interventions on dynamic balance performance in the CT and ST groups. FC represents forward composite latency during the Motor Control Test. TOESUP and TOESDOWN represent sway-energy scores during the Adaptation Test under toes-up and toes-down perturbations. YBT-N represents the normalized Y-Balance Test composite score, and YBT-D represents the dominant-limb Y-Balance Test score. The Closed-Eye In-Place Stepping Test represents the time required to reach the boundary of the testing circle with eyes closed. Values are expressed as mean ± standard deviation. Boxes represent the interquartile range, horizontal lines indicate medians, whiskers represent data distribution, and black dots indicate individual participant values. *Indicates a significant within-group pre–post difference (p < 0.05). ^#^Indicates a significant between-group difference at post-intervention (p < 0.05). CT, unstable-surface combined training group; ST, stable-surface comparison group; YBT, Y-Balance Test.

**Figure 5:**
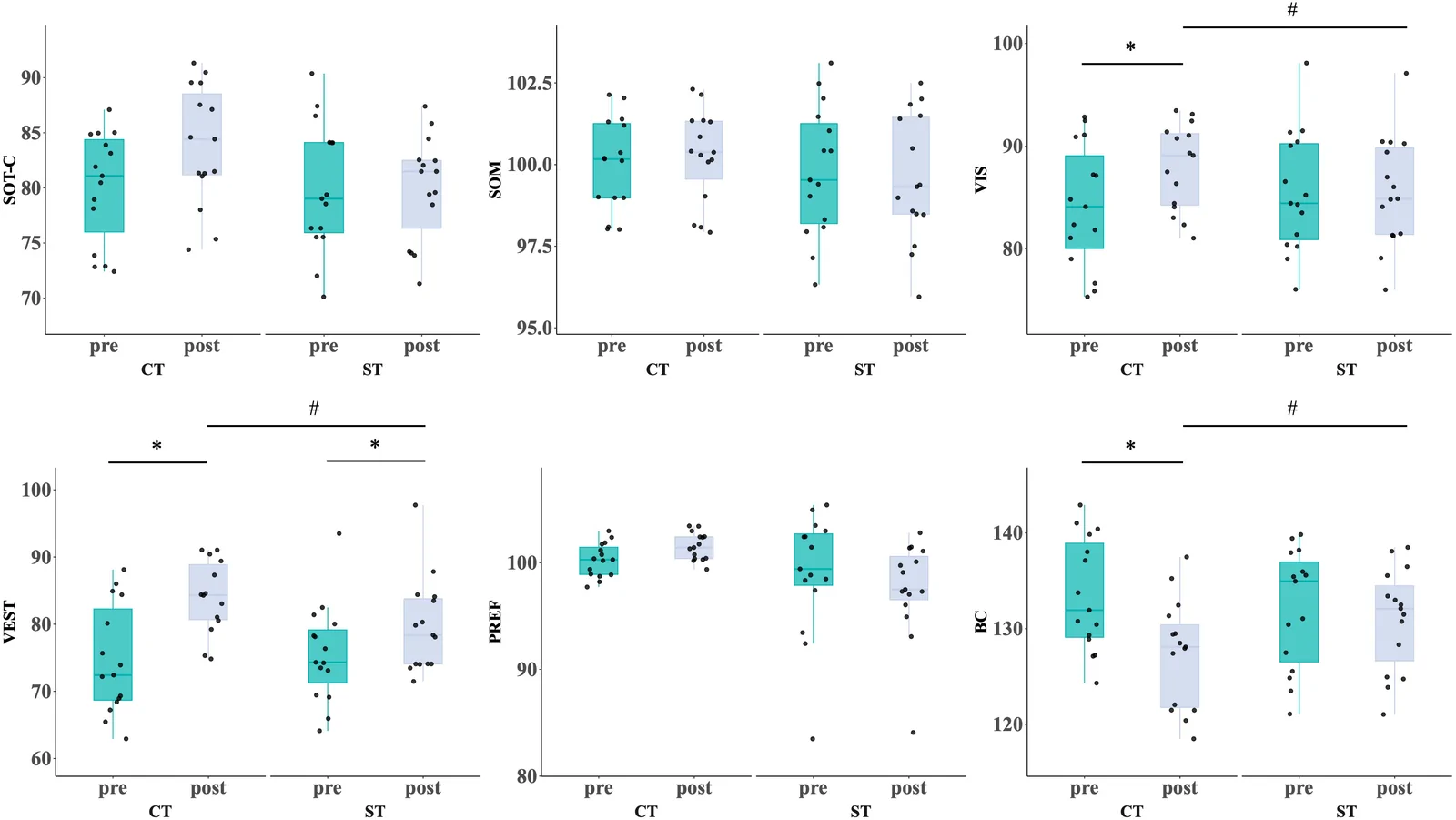
Changes in sensory-organization and motor-control outcomes before and after the 12-week intervention in the CT and ST groups. SOT-C represents the Sensory Organization Test composite score. SOM, VIS, VEST, and PREF represent the somatosensory, visual, vestibular, and visual-preference ratios, respectively. BC represents backward composite latency during the Motor Control Test. Values are expressed as mean ± standard deviation. Boxes represent the interquartile range, horizontal lines indicate medians, whiskers represent data distribution, and black dots indicate individual participant values. *Indicates a significant within-group pre-post difference (p < 0.05). #Indicates a significant between-group difference at post-intervention (p < 0.05). CT, unstable-surface combined training group; ST, stable-surface comparison group.

For dynamic-balance outcomes, significant main effects of time and significant group × time interactions were observed for VIS, VEST, toes-up adaptation, toes-down adaptation, YBT-N, YBT-D, and the Closed-Eye In-Place Stepping Test (all p < 0.05; **Table 5**; **Figures 4**, **5**). No significant time effects or group × time interactions were found for the SOT composite score, SOM, or PREF. Bonferroni-adjusted comparisons showed significant improvements in the CT group for VIS, VEST, toes-up adaptation, toes-down adaptation, YBT-N, YBT-D, and closed-eye stepping performance. The ST group showed significant improvements only in VEST and closed-eye stepping performance. These findings indicate broader improvements in sensory-related and dynamic postural-control outcomes in CT than in ST, although the SOT composite score did not change significantly.

**Table 5:** The dynamic balance assessment results for CT group and ST group before and after 12-week training.

Outcome	CT group			ST group			Time effect	Time × group interaction effect
	Pre	Post	Cohen’s d (95%CI)	Pre	Post	Cohen’s d (95%CI)		
SOT-C	80.10 ± 5.04	83.83 ± 5.42	-0.705 (-1.484, 0.074)	79.96 ± 5.90	79.91 ± 4.74	0.010 (-0.725, 0.744)	0.076	0.068
SOM	100.00 ± 1.43	100.25 ± 1.41	-0.145 (-0.753, 0.463)	99.78 ± 2.02	99.58 ± 1.97	0.118 (-0.489, 0.726)	0.932	0.401
VIS	84.18 ± 6.01	87.96 ± 4.17^*#^	-0.698 (-1.190, -0.207)	85.50 ± 5.88	85.57 ± 5.40	-0.454 (-1.493, 0.585)	0.002	0.003
VEST	74.67 ± 8.15	84.14 ± 5.26^*#^	-1.350 (-2.041, -0.658)	75.60 ± 7.34	76.69 ± 7.00^*^	-0.584 (-1.104, -0.063)	<0.001	0.003
PREF	100.24 ± 1.59	101.53 ± 1.28	-0.339 (-0.771, 0.092)	99.00 ± 5.73	97.53 ± 4.59	0.386 (-0.051, 0.823)	0.824	0.184
BC	133.54 ± 5.91	127.42 ± 5.62^*#^	1.056 (0.259, 1.853)	132.08 ± 6.25	130.98 ± 5.37	0.190 (-0.508, 0.889)	0.002	0.022
FC	133.32 ± 6.10	128.83 ± 5.68^*^	0.836 (0.138, 1.534)	132.70 ± 4.75	130.63 ± 4.79	0.386 (-0.256, 1.027)	<0.001	0.169
Toes up	76.77 ± 7.70	60.06 ± 7.54^*#^	2.038 (1.194, 2.881)	76.80 ± 9.36	70.84 ± 8.08	0.727 (0.282, 1.172)	<0.001	<0.001
Toes down	51.04 ± 5.34	42.44 ± 5.17^*#^	1.611 (0.837,2. 385)	52.62 ± 5.65	48.68 ± 5.19	0.737 (0.177, 1.296)	<0.001	0.001
YBT-N	88.17 ± 5.04	95.53 ± 4.49^*#^	-1.387 (-2.093, -0.681)	88.97 ± 6.66	91.20 ± 4.76	-0.420 (-0.924, 0.084)	<0.001	<0.001
YBT-D	92.86 ± 8.34	97.72 ± 6.97^*#^	-0.731 (-1.075, -0.387)	92.95 ± 5.96	94.14 ± 4.85	-0.179 (-0.395, 0.037)	<0.001	<0.001
Closed-Eye In-Place Stepping Test (s)	15.76 ± 3.30	33.73 ± 4.37^*#^	-4.192 (-6.828, -2.997)	14.96 ± 3.38	18.16 ± 3.28^*^	-0.877 (-1.542, -0.212)	<0.001	<0.001

^*^Statistically significant difference between pre- and post-test, P < 0.05.

^#^Statistically significant difference between CT group and ST group, P < 0.05.

### Change-of-direction performance

**Figure 6:**
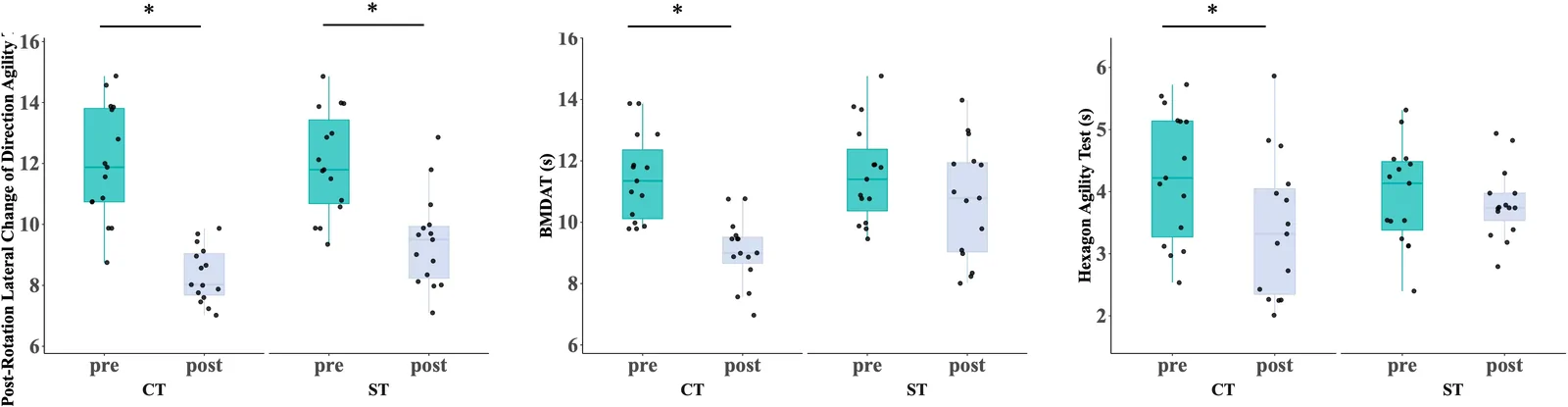
Changes in preplanned change-of-direction performance before and after the 12-week intervention in the CT and ST groups. The Post-Rotation Lateral Change-of-Direction Test, Ballet Multi-Directional Change-of-Direction Test (BMDAT), and Hexagon Agility Test were used to assess ballet-related and general change-of-direction performance. Completion time is expressed in seconds, with lower values indicating better performance. Values are expressed as mean ± standard deviation. Boxes represent the interquartile range, horizontal lines indicate medians, whiskers represent data distribution, and black dots indicate individual participant values. *Indicates a significant within-group pre-post difference (p < 0.05). #Indicates a significant between-group difference at post-intervention (p < 0.05). CT, unstable-surface combined training group; ST, stable-surface comparison group; BMDAT, Ballet Multi-Directional Change-of-Direction Test.

Significant main effects of time were observed for the Post-Rotation Lateral Change-of-Direction Test, BMDAT, and Hexagon Test (all p < 0.010; **Table 6**; **Figure 6**), indicating an overall improvement in change-of-direction performance following the intervention. However, no significant group × time interaction was observed for the Post-Rotation Lateral Change-of-Direction Test (p = 0.156), BMDAT (p = 0.054), or Hexagon Test (p = 0.054). Therefore, the magnitude of improvement did not differ significantly between the CT and ST groups, and the results do not support an additional benefit of unstable-surface balance training for change-of-direction performance.

**Table 6:** The change of direction assessment results for CT group and ST group before and after 12-week training.

Outcome	CT group			ST group			Time effect	Time × group interaction effect
	Pre	Post	Cohen’s d (95%CI)	Pre	Post	Cohen’s d (95%CI)		
Post-Rotation Lateral Change of Direction Agility Test (s)	12.00 ± 1.89	8.35 ± 0.91^*^	2.352 (1.098, 3.605)	12.01 ± 1.72	9.43 ± 1.52^*^	1.665 (0.568, 2.761)	<0.001	0.156
BMDAT (s)	11.45 ± 1.42	9.05 ± 1.07^*^	1.571 (0.447, 2.695)	11.57 ± 1.61	10.70 ± 1.90	0.566 (-0.422, 1.554)	<0.001	0.054
Hexagon Agility Test (s)	4.27 ± 1.06	3.42 ± 1.14^*^	0.917 (0.098, 1.736)	3.94 ± 0.81	3.81 ± 0.57	0.148 (-0.600, 0.896)	0.010	0.054

^*^Statistically significant difference between pre- and post-test, P < 0.05.

^#^Statistically significant difference between CT group and ST group, P < 0.05.

## Discussion

This study examined whether adding unstable-surface balance training to a resistance training program would improve balance and change-of-direction performance in male college ballet dancers to a greater extent than performing the same balance exercises on stable surfaces. The main findings were that the unstable-surface group demonstrated broader improvements in static and dynamic balance, whereas both groups improved in agility-related outcomes over time. Both groups improved in change-of-direction performance over time. However, because none of the three outcomes showed a significant group × time interaction, the present findings do not demonstrate an additional change-of-direction benefit of unstable-surface balance training beyond the common resistance-training and balance-training program. Overall, these findings suggest that unstable-surface balance training may provide additional benefits when used as an adjunct to resistance training in college ballet dancers. This pattern suggests that the added surface challenge may be most useful for ballet-relevant postural-control outcomes rather than for producing uniform improvements across all performance domains.

CT produced significant improvements across a wide range of balance indicators. Under both eyes-open and eyes-closed conditions, sway amplitude and velocity were reduced, indicating more efficient postural regulation. Improvements in the anteroposterior direction (Y-axis) in both CT and ST suggest that resistance training may enhance sagittal-plane control, likely through improvements in lower-limb and trunk strength (Gonçalves et al., 2020). This interpretation is consistent with Langeard et al (Langeard et al., 2020), who reported that strength training can improve general postural stability, particularly in forward-backward sway, where lower-limb extensor strength plays an important role. The CT group improved across all mediolateral sway measures under both eyes-open and eyes-closed conditions, whereas the ST group showed improvements only in the eyes-closed mediolateral measures. This pattern suggests that unstable-surface training produced broader, rather than exclusive, improvements in mediolateral postural control. This pattern may be related to the greater postural challenge imposed by unstable-surface exercises, which require continuous corrective adjustments to maintain equilibrium. Previous reviews have suggested that balance-oriented training can improve postural sway and functional balance, with potential contributions from enhanced movement regulation and neuromuscular control (Behm et al., 2015; Zech et al., 2010). In the present study, the larger improvement in mediolateral control in the CT group is therefore consistent with the view that unstable-surface training may provide a more specific stimulus for lateral stability, an ability that is highly relevant to single-leg support and rotational movements in ballet.

The absence of significant change in X-axis measures in the ST group may be related to the current resistance training protocol, which focused primarily on sagittal-plane movements and may not have sufficiently targeted lateral support strength. McCormick et al (McCormick et al., 2014). noted that agility and lateral control depend on specific strength capacities in the frontal plane, which are often undertrained in traditional resistance programs. In addition, stable-surface balance exercises may provide a smaller challenge to mediolateral control, which could explain their reduced effectiveness for this aspect of postural stability. The improved visual and vestibular ratio scores in the CT group may also reflect better performance under sensory-challenging balance conditions. Postural control depends on the flexible integration and weighting of visual, vestibular, and somatosensory information according to task demands (Assländer and Peterka, 2014; Horak, 2006). From this perspective, the CT group’s improvements are compatible with more effective balance regulation in situations where sensory information must be rapidly adjusted, such as turning, single-leg support, or performance under reduced visual stability. This adaptation is particularly relevant for ballet dancers, who often perform under conditions of reduced visual stability, such as during rapid turns or in dim lighting. This interpretation is consistent with Hamed et al (Hamed et al., 2018), who highlighted that unstable training conditions can facilitate sensory reweighting by requiring greater reliance on vestibular and somatosensory inputs. Although both groups showed some improvement in balance tests, the more consistent and multidimensional gains in CT support the view that functional balance outcomes are influenced not only by strength development but also by the context in which strength is applied.

With respect to agility and change-of-direction performance, both groups improved over time, indicating that the overall training program enhanced movement performance. Because both groups completed the same resistance training protocol, part of this improvement likely reflects gains in strength, movement control, and general physical preparedness. Although the CT group showed clearer within-group improvements in the post-rotation lateral change-of-direction test, the BMDAT, and the Hexagon Agility Test, the present results did not consistently demonstrate significant group × time interactions across all agility outcomes. Accordingly, these findings should be interpreted cautiously: unstable-surface balance training may have contributed to additional improvements in selected ballet-relevant directional tasks, but generalized superiority of CT over ST for agility cannot be concluded. This interpretation remains consistent with previous work suggesting that agility is influenced not only by strength but also by balance and neuromuscular coordination (Sanchez-Sanchez et al., 2022; Sañudo et al., 2019). The interpretation of the ballet-specific agility outcomes should also be considered within a measurement-validity framework. The post-rotation lateral COD test and the BMDAT were intended to improve content and ecological relevance by embedding speeded directional changes within ballet-specific movement constraints, including rotation, pas de bourrée, arabesque, relevé, turnout, head control, and pointe-shoe conditions. In dancers, physical conditioning is also closely linked to technical execution and aesthetic requirements rather than being an isolated athletic capacity (Long et al., 2021). Therefore, the observed improvements in these tests should be interpreted as preliminary evidence of improved ballet-related change-of-direction performance under dance-constrained conditions, rather than as direct evidence of improved overall ballet performance. Future studies should formally examine construct and criterion validity by relating these tests to expert-rated ballet technique, choreographic performance quality, biomechanical movement-quality indicators, and dance-specific functional outcomes.

A principal strength and novel feature of the present study was the matched active-comparison design. Both groups completed identical resistance training and identical balance exercises with matched exercise order, volume, external load, contact time, and recovery intervals, allowing the specific contribution of support-surface instability to be examined. From a practical perspective, the findings suggest that unstable-surface balance exercises may be useful when the primary conditioning objective is to improve mediolateral and sensory-related postural control in male college ballet dancers. However, because none of the change-of-direction outcomes showed a significant group × time interaction, unstable-surface training should not be expected to provide a generalized advantage across all aspects of movement performance.

Several limitations should be considered when interpreting the findings. First, the sample size was relatively small, and the cohort consisted exclusively of male college ballet dancers. Therefore, the findings should not be generalized directly to female ballet dancers or to dancers of other ages and performance levels. The present design also did not allow potential sex-related differences in baseline postural control or responsiveness to unstable-surface balance training to be examined. Future studies should recruit adequately powered female and mixed-sex cohorts and investigate whether intervention responses differ by sex. Second, the study was not registered in a publicly accessible trial registry. Although the intervention procedures, outcome measures, and statistical analyses were determined before data analysis, the absence of trial registration limits transparency and should be considered when evaluating the methodological rigor of the study. Third, although the two ballet-specific agility tests were designed to improve ecological relevance by embedding COD tasks within rotation, pas de bourrée, arabesque, relevé, turnout, and pointe-shoe constraints, their validation remains preliminary. The pilot ICC values support test-retest reliability, and the task components provide content-based justification for ballet relevance, but formal construct validation against expert-rated ballet performance, biomechanical quality indicators, or other dance-performance outcomes has not yet been established. These outcomes should therefore be interpreted as exploratory indicators of ballet-related COD performance rather than definitive measures of overall ballet performance. Fourth, the active-comparison design allowed comparison of unstable- and stable-surface balance training under the same resistance training background, but it did not isolate the independent effects of resistance training from those of the added balance intervention. Fifth, the study did not include neuromuscular, biomechanical, or sensory-processing measures, which limits mechanistic interpretation. Finally, training adherence, adverse events, and longer-term retention of training effects were not examined and should be addressed in future research.

## Conclusion

This study showed that adding unstable-surface balance training to resistance training produced broader improvements in static and dynamic balance in male college ballet dancers than performing the same balance exercises on stable surfaces. Both groups improved in change-of-direction performance over time; however, no significant between-group differences in the magnitude of change were observed. Therefore, the additional benefit of unstable-surface training appears to be specific mainly to selected postural-control outcomes rather than change-of-direction performance.

## Data Availability

The original contributions presented in the study are included in the article/supplementary material. Further inquiries can be directed to the corresponding author.
